# Optical tomographic imaging discriminates between disease-modifying anti-rheumatic drug (DMARD) and non-DMARD efficacy in collagen antibody-induced arthritis

**DOI:** 10.1186/ar3038

**Published:** 2010-05-28

**Authors:** Jeffrey D Peterson, Timothy P LaBranche, Kristine O Vasquez, Sylvie Kossodo, Michele Melton, Randall Rader, John T Listello, Mark A Abrams, Thomas P Misko

**Affiliations:** 1 VisEn Medical Inc, 45 Wiggins Avenue, Bedford, MA 01730, USA; 2 Pfizer Global Research & Development, 700 Chesterfield Parkway West, Chesterfield, St Louis, MO 63017, USA

## Abstract

**Introduction:**

Standard measurements used to assess murine models of rheumatoid arthritis, notably paw thickness and clinical score, do not align well with certain aspects of disease severity as assessed by histopathology. We tested the hypothesis that non-invasive optical tomographic imaging of molecular biomarkers of inflammation and bone turnover would provide a superior quantitative readout and would discriminate between a disease-modifying anti-rheumatic drug (DMARD) and a non-DMARD treatment.

**Methods:**

Using two protease-activated near-infrared fluorescence imaging agents to detect inflammation-associated cathepsin and matrix metalloprotease activity, and a third agent to detect bone turnover, we quantified fluorescence in paws of mice with collagen antibody-induced arthritis. Fluorescence molecular tomographic (FMT) imaging results, which provided deep tissue detection and quantitative readouts in absolute picomoles of agent fluorescence per paw, were compared with paw swelling, clinical scores, a panel of plasma biomarkers, and histopathology to discriminate between steroid (prednisolone), DMARD (p38 mitogen-activated protein kinase (MAPK) inhibitor) and non-DMARD (celecoxib, cyclooxygenase-2 (COX-2) inhibitor) treatments.

**Results:**

Paw thickness, clinical score, and plasma biomarkers failed to discriminate well between a p38 MAPK inhibitor and a COX-2 inhibitor. In contrast, FMT quantification using near-infrared agents to detect protease activity or bone resorption yielded a clear discrimination between the different classes of therapeutics. FMT results agreed well with inflammation scores, and both imaging and histopathology provided clearer discrimination between treatments as compared with paw swelling, clinical score, and serum biomarker readouts.

**Conclusions:**

Non-invasive optical tomographic imaging offers a unique approach to monitoring disease pathogenesis and correlates with histopathology assessment of joint inflammation and bone resorption. The specific use of optical tomography allowed accurate three-dimensional imaging, quantitation in picomoles rather than intensity or relative fluorescence, and, for the first time, showed that non-invasive imaging assessment can predict the pathologist's histology inflammation scoring and discriminate DMARD from non-DMARD activity.

## Introduction

Rheumatoid arthritis (RA) is a chronic destructive inflammatory disease of the joints. Although the disease pathogenesis remains unclear, there is significant evidence implicating T cells and B cells in the early initiating steps of disease and innate immunity in its chronic, slow progression [1]. Both genetic and environmental factors contribute to the development of RA [2], and the disease shows a steady progression of synovial hyperplasia and neovascularization, mixed mononuclear and granulocytic cellular infiltration, damage to articular cartilage, bone remodeling, and proliferation of both synovial and extraarticular fibroblasts [1,3]. This manifests clinically as swelling, erythema, and pain, and can progress to decreased bone density and obvious joint architecture changes.

Of current importance in the development of anti-arthritic drugs is the ability to discriminate between disease-modifying anti-rheumatic drugs (DMARDs), which affect arthritis pathogenesis and progression, and non-DMARDs, which may show palliative effects and symptom relief in the absence of affecting disease progression. DMARD treatments include antiproliferative drugs (for example, leflunamide and methotrexate) or cytotoxic drugs (azathioprine) as well as agents that interfere with TNFα, such as anti-TNF biologics (adalumimab, etanercept, infliximab). Inhibitors of p38 mitogen-activated protein kinase (MAPK) have also been shown to reduce TNF levels and affect disease pathogenesis in animal models of RA [4-8], with some more modest effects in patients showing DMARD efficacy [4,9] limited by dose-dependent toxicity. Whereas p38 MAPK inhibitors significantly decrease underlying inflammation and bone destruction, cyclooxygenase-2 (COX-2) inhibitors, such as celecoxib, and other nonsteroidal anti-inflammatory drugs (NSAIDs) are better at providing symptom relief than at altering disease progression [10,11].

A variety of rodent arthritis models have been used to study arthritis disease progression and the impact of promising new therapies [12]. These models include the current gold standard approaches using type II collagen-induced arthritis in both the mouse and rat, and have been used extensively for benchmarking novel therapies while being routinely validated against current standards of care (methotrexate and prednisolone). Their utility is limited, however, as mouse collagen-induced arthritis models require specific disease-susceptible inbred mouse strains (that is, DBA/1 and B10.RIII) in order to develop arthritis, placing a heavy emphasis on the early inductive phase of disease. In contrast, newer models that bypass the cognate immunity step in disease induction by using inducing antibodies to trigger chronic disease, such as the collagen antibody-induced arthritis (CAIA) model, provide a more straightforward and rapid means of producing disease pathology that is both independent of the mouse strain and can be used with transgenic or knockout mice [13-16]. Although the mouse CAIA model does not have the extensive history and therapeutic validation of the collagen-induced arthritis model, there is growing support for the relevance of autoantibodies in mouse arthritis [17-23] and in human arthritis [24-27], and there is particular evidence suggesting the importance of autoantibodies at disease onset [28].

Regardless of the particular rodent model used to study disease mechanisms, current non-invasive standard readouts of disease severity - such as paw thickness/volume or clinical score grades - do not provide a quantitative biological readout of the cellular/tissue-specific processes contributing to disease progression. For instance, paw thickness uses dimensional changes in the paw as a surrogate marker for underlying edema and inflammation, while clinical score assessment is a subjective assessment of paw swelling and erythema. Although these readouts can be useful measures of disease severity, they emphasize the edema component of disease rather than the underlying synovial proliferation, inflammatory cell infiltration and, osteoclast-mediated bone resorption. Paw swelling or clinical scores therefore do not discriminate well between DMARD and non-DMARD treatments such as NSAIDs. For instance, the non-DMARD anti-inflammatory COX-2 inhibitors (a type of NSAID) routinely demonstrate efficacy in a variety of rodent arthritis models, as determined by paw swelling/clinical score [5-7,12,29,30]. Because of this, there is significant reliance upon (terminal) histopathology to discriminate DMARD activity from NSAID activity when assessing new drugs.

In the present article, we build upon recent advances in optical tomographic imaging and near-infrared (NIR) agents [31-36] to test the hypotheses that biological imaging of molecular optical biomarkers of inflammation and bone turnover would provide superior non-invasive (nonterminal), quantitative readouts for underlying disease pathology, and that - when used in combination with optical tomographic imaging - the CAIA model should provide robust and quick discrimination between DMARD and non-DMARD treatments.

Our studies illustrate the ability of three-dimensional fluorescence molecular tomographic (FMT) quantification to discriminate between DMARD and non-DMARD effects. For instance, neither clinical score, paw thickness, nor multiple plasma biomarkers could differentiate between a p38 MAPK inhibitor and the COX-2 inhibitor celecoxib, while FMT quantification using NIR agents to detect cathepsin, matrix metalloprotease (MMP), or bone resorption activity yielded a clear discrimination between these two classes of treatment. FMT results agreed well with histopathologic scoring of inflammation, and both FMT and histology measures identified clear deficiencies in clinical score and paw-swelling assessments of disease. Optical tomographic imaging of disease biology offers a non-invasive, nonterminal measure of disease that strongly correlates with the underlying pathology of the CAIA model and allows for discriminating between DMARD and non-DMARD therapeutics.

## Materials and methods

### Experimental animals

Specific pathogen-free female BALB/c mice (4 to 6 weeks of age, 18 to 20 g) were obtained from Charles River (Wilmington, MA, USA) and were housed in a controlled environment (72°F; 12 h:12 h light-dark cycle) under specific-pathogen free conditions with water and food provided *ad libitum*. All experiments were performed in accordance with VisEn IACUC guidelines for ethical animal care and use.

### Therapeutic studies with the collagen antibody-induced arthritis animal model

BALB/c mice were injected intravenously with 4 mg arthrogen-collagen-induced arthritis monoclonal antibody cocktail (Clones D1, F10, A2 and D8 to collagen type II; Chemicon, Temecula, CA, USA), according to the manufacturer's instructions. Measurable morphological changes were determined by paw thickness measurement using a digital Vernier caliper (VWR, West Chester, PA, USA) on days 4, 6, and 8. Observational clinical scores (scale from 0 to 3) were also made based upon the following criteria of redness and swelling: 0 = no swelling or redness (normal paws), 1 = swelling and/or redness in one digit or in the ankle, 2 = swelling and/or redness in one or two digits and ankle, and 3 = entire paw is swollen or red.

Beginning on day 3 post antibody cocktail injection (prior to signs of disease), cohorts of CAIA mice (n = 12 per group) were treated daily (8 or 15 days) with either prednisolone (10 mg/kg per oral, twice daily), a p38 MAPK inhibitor (SD0006; 15 mg/kg per oral, twice daily), and celecoxib (15 mg/kg per oral, twice daily). Two additional groups, healthy mice (n = 12) and arthritic mice (n = 12), received vehicle treatment only (0.5% aqueous methyl cellulose and 0.025% Tween-80) and served as controls.

### Fluorescent agents for the detection of inflammation

Three commercially available imaging agents (VisEn Medical Inc., Bedford, MA, USA) were used to measure disease and therapeutic efficacy in CAIA. For assessing the inflammatory infiltrate, two NIR protease-activatable agents were used, one activated by cathepsins (ProSense750) and the other activated by a family of MMPs (MMPSense680), including MMP-3, MMP-9, and MMP-13. These agents were administered via intravenous route (2 nmol (fluorophore) in 150 μl saline) in all imaging studies. A third NIR imaging agent that detects changes in bone associated with disease (OsteoSense680) was used to image and quantify bone loss. For MMPSense680 and OsteoSense680, the 2 nmol dose of fluorophore corresponds to 2 nmol substrate or pamidronate, respectively. For ProSense750, the 2 nmol dose of fluorophore corresponds to ~0.1 nmol substrate.

### Imaging arthritis disease progression

CAIA and control mice were injected intravenously with ProSense750 or MMPSense680 on day 7 following injection of collagen antibody cocktail. OsteoSense680 was injected in additional studies on both day 7 and day 14. At the time of imaging (24 h post agent injection), mice were anesthetized using an intraperitoneal injection of ketamine (100 mg/kg) and xylazine (20 mg/kg). CAIA and control mice were then imaged with the FMT 2500™ fluorescence tomography *in vivo *imaging system (VisEn Medical) using fluorescence tomographic scanning capabilities as described previously [37]. Briefly, the anesthetized mice were carefully positioned in a prone position in the imaging cassette. Both hind paws were elevated on a resin block (designed to mimic optical scattering and absorption properties of the mouse's body) to allow larger tomographic scanning fields for simultaneous imaging of both paws. A NIR laser diode transilluminated the hindpaws, with signal detection occurring via a thermoelectrically cooled charge-coupled device camera placed on the opposite side of the imaged animal. Appropriate optical filters allowed collection both of fluorescence and excitation datasets, the entire imaging acquisition requiring 4 to 5 minutes per mouse.

### Fluorescence molecular tomographic reconstruction and analysis

The collected fluorescence data were reconstructed by FMT 2500 system software (TruQuant™; VisEn Medical) for the quantification of the fluorescence signal within the paws. Three-dimensional regions of interest were drawn to encompass each foot and subregions of the foot. A threshold was applied identically to all animals equal to twice the mean paw fluorescence (nanomolar) of the control, nonarthritic mice to minimize low-intensity, background fluorescence. The total amount of ankle, midfoot, toes or total paw fluorescence (in picomoles) was automatically calculated relative to internal standards generated with known concentrations of appropriate NIR dyes. For visualization and analysis purposes, the FMT 2500 system software provided three-dimensional images and tomographic slices.

### Histopathology

The right ankle from each animal was fixed in 10% neutral buffered formalin for 24 hours at 20°C, followed by decalcification in Immunocal™ (Decal Chemical Corporation, Tallman, NY, USA) for 7 days at 20°C. Decalcified joints were then paraffin embedded, sectioned twice (4 μm each), and stained with H & E for general evaluation or toluidine blue for specific assessment of cartilage changes. The ankles were evaluated via histopathology and scored for inflammation, cartilage damage, pannus and bone resorption according to previously published criteria [38].

For inflammation, scores were as follows: 0 = normal, 1 = minimal infiltration of inflammatory cells in the synovial and/or periarticular tissues, 2 = mild infiltration with mild edema, 3 = moderate infiltration (including joint space) with moderate edema, 4 = marked infiltration with marked edema, and 5 = severe infiltration with severe edema.

For cartilage damage, scores were as follows: 0 = normal, 1 = loss of toluidine blue staining with no chondrocyte degeneration/loss and/or matrix disruption, 2 = loss of toluidine blue staining with minimal chondrocyte degeneration/loss and/or mild matrix disruption in some affected joints, 3 = loss of toluidine blue staining with moderate chondrocyte loss and obvious (depth to deep zone) matrix loss in affected joints, 4 = loss of toluidine blue staining with marked (depth to tide mark) chondrocyte and matrix loss, and 5 = loss of toluidine blue staining with severe (depth to subchondral bone) chondrocyte loss and matrix loss in affected joints.

For bone resorption, scores were as follows: 0 = normal, 1 = minimal (small areas of resorption in the medullary trabecular or cortical bone, not readily apparent on low magnification, and rare osteoclasts), 2 = mild (increasing areas of resorption in medullary trabecular or cortical bone, not readily apparent on low magnification, with osteoclasts more numerous), 3 = moderate (obvious resorption of the medullary trabecular and cortical bone, without full-thickness defects, lesion apparent on low magnification, and osteoclasts more numerous), 4 = marked (full-thickness defects in the cortical bone, marked loss of medullary trabecular bone, numerous osteoclasts), and 5 = severe (full-thickness defects in the cortical bone, severe loss of medullary trabecular bone).

### Immunoassay analysis of plasma biomarkers

Plasma MMP-3, a soluble marker for joint pathology, was quantified by the R&D System (Minneapolis, MN, USA) Quantikine mouse MMP-3 (total) Immunoassay (catalog number MMP300) according to the manufacturer's instructions. Plasma cytokines and chemokines - eotaxin, granulocyte colony-stimulating factor (G-CSF), granulocyte-macrophage colony-stimulating factor, GRO/KC, IFNγ, leptin, IL-1α, IL-1β, IL-2, IL-4, IL-5, IL-6, IL-9, IL-10, IL-12p70, IL-13, IL-17, IL-18, IP-10, MCP-1, MIP-1β, RANTES, TNFα and vascular endothelial growth factor - were assessed using a multiplex Luminex-based assay from Millipore (catalog number MPXMCYTO-70K-PMX24; Billerica, MA USA) with the addition of 1× Complete^® ^protease inhibitor cocktail (catalog number 11697498001; Roche, Indianapolis, IN, USA).

### Statistical analysis

Data are presented as the mean ± standard error of the mean. Significance analysis of *in vivo *paw fluorescence was conducted using a two-tailed unpaired Student *t *test when two groups were analyzed or a one-tailed analysis of variance Scheffe multiple-comparison post test. *P *< 0.05 was considered significant.

## Results

### Standard measures of CAIA progression (paw edema and clinical scoring) do not separate DMARD from non-DMARD treatments

To assess whether mouse CAIA can be used to effectively discriminate between DMARD and non-DMARD treatments, BALB/c mice were injected with a cocktail of anti-collagen type II antibodies, boosted with lipopolysaccharide on day 3, and treatments were initiated on day 4. Prednisolone (as a steroid), a p38 MAPK inhibitor [8] (as a DMARD treatment), and the non-DMARD COX-2 inhibitor celecoxib (an NSAID) were used to characterize different classes of treatments. Animals were treated throughout the study until the peak of disease on day 8. Nondiseased controls, vehicle, and treated mice were assessed for changes in paw thickness and clinical score on days 4, 6, and 8.

**Figure 1:**
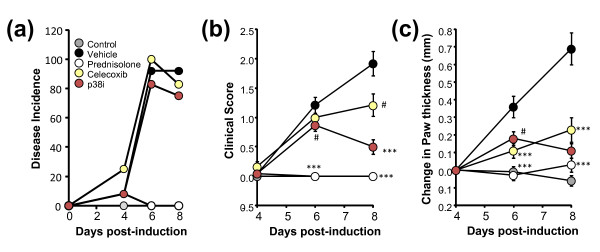
**Disease incidence, clinical score, and paw thickness readouts of treated and untreated CAIA mic**. **(a) **Disease incidence in control and treated collagen antibody-induced arthritis (CAIA) mice (n = 12 mice per group), defined as any animal showing a clinical score ≥1 in at least one paw. **(b) **Average clinical score values of all paws for control and treated CAIA mice (n = 12 mice per group). **(c) **Average changes in paw thickness from day 4 to day 8 for control and treated CAIA mice. Study is representative of three separate experiments. ^#^*P *< 0.05, ***P < 0.0001.

The disease incidence (Figure 1a) was 92% in the vehicle group, with 90% of individual arthritic paws showing a clinical score ≥1 by day 8. Treatment groups showed comparable kinetics and incidence of disease. Prednisolone treatment, as a strong positive control, effectively ablated the clinical score (Figure 1b) and paw swelling (Figure 1c) endpoints as early as day 6 post disease induction, maintaining this effect through to the end of the study. The p38 MAPK inhibitor showed mild, but significant, inhibition of clinical score at day 6, with an increase in efficacy by day 8; and celecoxib showed a similar trend, albeit with less overall effect by day 8. All treatments showed highly significant inhibition of paw swelling (Figure 1c). The clinical scoring and paw swelling readouts thus poorly discriminated between the different types of treatments.

### Histopathology and biomarker assessment of CAIA and treatment efficacy

**Figure 2:**
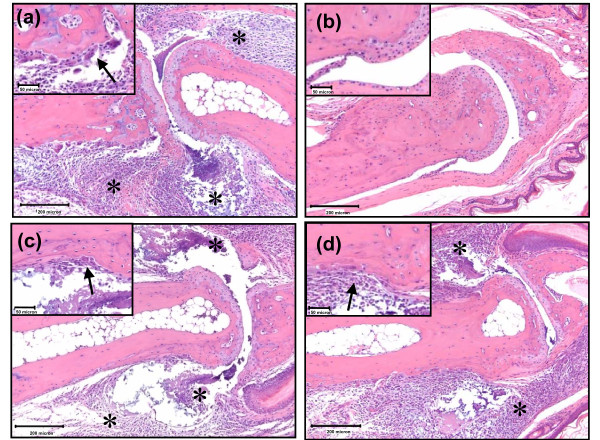
**Histology of treated and untreated collagen antibody-induced arthritis mice**. **(a) **Collagen antibody-induced arthritis (CAIA)/vehicle mouse interphalangeal joint (10× magnification) showing expansion of synovial and extraarticular tissues, as well as the joint spaces, by edema and inflammatory cell infiltrates (asterisks). Inset: higher magnification (40×) view of osteoclast-mediated bone resorption (arrow). **(b) **Prednisolone-treated mouse interphalangeal joint (10× magnification) with normal synovial tissue and cartilage. Inset: high magnification view (40×) showing absence of bone resorption. **(c) **Celecoxib-treated mouse interphalangeal joint (10× magnification) also with inflammatory cells in the joint capsule, synovium, and joint space (asterisks). Inset: higher magnification (40×) view of osteoclast-mediated bone resorption (arrow). **(d) **p38 mitogen-activated protein kinase (MAPK) inhibitor-treated mouse interphalangeal joint (10× magnification) with mildly decreased edema and inflammation in the joint space (asterisks) as compared with vehicle and celecoxib groups. Inset: higher magnification (40×) view showing inflammation (arrow) but minimal apparent osteoclast-mediated bone resorption. Histology assessment is representative of two separate experiments.

Histopathologic assessment of vehicle-treated mice revealed edema and inflammatory cell influx (general inflammation) in the synovial tissues, joint spaces and extra-articular soft tissues, in addition to mild articular cartilage damage, mild osteoclast-mediated bone resorption, and extraarticular fibroplasia; no appreciable pannus formation was observed in this model (Figure 2a). These results were as expected for this acute model of antibody-induced arthritis. Prednisolone treatment ablated all microscopic evidence of disease (Figure 2b), whereas celecoxib showed no reduction in edema, inflammation or bone resorption (Figure 2c). The p38 MAPK inhibitor decreased edema and inflammatory cell infiltration, particularly within the joint space, compared with vehicle-treated and celecoxib-treated groups (Figure 2d).

**Figure 3:**
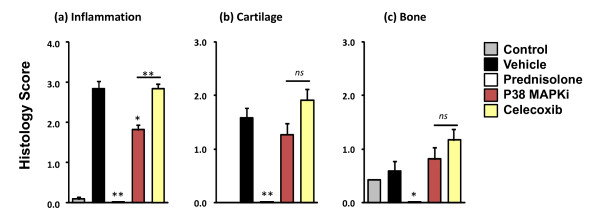
**Histopathology scoring**. Hindpaw tissues (n = 12 per group, one paw from each mouse) from the study represented in Figure 1 were processed for histopathology assessment. **(a) **Histopathology severity scores for general inflammation. **(b) **Histopathology severity scores for cartilage damage. **(c) **Histopathology severity scores for osteoclast-mediated bone resorption. Histology scoring results are representative of two separate experiments. *P < 0.01, **P < 0.001; *ns*, not significant. MAPKi, mitogen-activated protein kinase inhibitor.

There was a statistically significant decrease in general inflammation histopathology scores in both the prednisolone and p38 MAPK inhibitor-treated groups, but not in the celecoxib-treated group (Figure 3a). Cartilage damage and osteoclast-mediated bone resorption was mild in the vehicle-treated group (Figure 3b and 3c, respectively), as expected in this acute and rapid model. Most notably, statistically significant decreases were only observed in prednisolone-treated mice.

**Figure 4:**
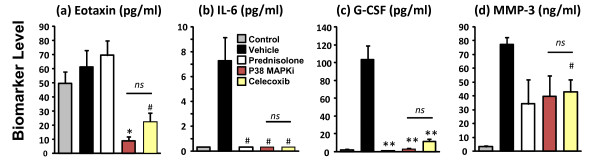
**Plasma biomarker measurement**. Day 8, peak disease plasma samples from the study represented in Figure 1 (n = 12 per group) were analyzed for a variety of biomarker changes as described in Materials and methods. **(a) **Quantification of plasma eotaxin levels. **(b) **Quantification of IL-6 levels. **(c) **Quantification of granulocyte colony-stimulating factor (G-CSF) levels. **(d) **Quantification of matrix metalloproteinase MMP-3 levels. ^#^*P *< 0.05, *P < 0.01, **P < 0.001; *ns*, not significant. MAPKi, mitogen-activated protein kinase inhibitor.

Plasma samples from control, vehicle, and treated mice were analyzed for levels of MMP-3 as well as a variety of cytokines and chemokines (eotaxin, G-CSF, granulocyte-macrophage colony-stimulating factor, GRO/KC, IFNγ, leptin, IL-1α, IL-1β, IL-2, IL-4, IL-5, IL-6, IL-9, IL-10, IL-12p70, IL-13, IL-17, IL-18, IP-10, MCP-1, MIP-1β, RANTES, TNFα and vascular endothelial growth factor). The majority of the plasma cytokine/chemokine panel showed either very low levels or no appreciable pattern of change (data not shown). We detected significant plasma elevations of only IL-6, G-CSF, and MMP-3 at day 8 (peak disease) in the mouse CAIA model when compared with naïve animals (Figure 4b to 4d). Eotaxin plasma levels, although showing no increase in vehicle-treated animals, decreased significantly with celecoxib and p38 MAPK inhibitor treatments, but not with prednisolone treatment (Figure 4a). IL-6 and G-CSF biomarkers increased in CAIA and were significantly decreased by all treatments to a similar extent (Figures 4b, c). In contrast, disease-related increases in plasma MMP-3 were decreased ~50% by all treatments with minimal statistical significance. Plasma levels of IL-6, G-CSF, and MMP-3 at day 8 did not discriminate between DMARD and non-DMARD treatments (Figure 4b to 4d), and no disease-related elevations of these biomarkers were observed on day 15 of disease (data not shown).

### Tomographic imaging provides a clear discrimination of arthritis disease severity

**Figure 5:**
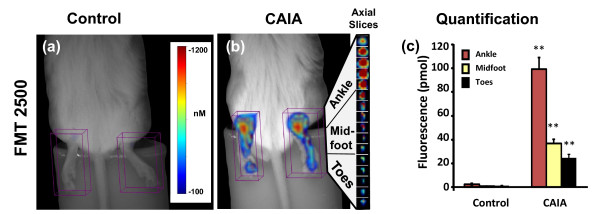
**Tomographic imaging of arthritis in collagen antibody-induced arthritis mice**. Arthritic and control BALB/c mice (n = 12 mice per group) were injected intravenously with ProSense750 on day 7 and imaged by FMT2500™ using tomographic scanning capabilities. **(a) **Near-infrared (NIR) tomographic imaging of the hind paws of a BALB/c control mouse. **(b) **NIR tomographic imaging of the hind paws of an arthritic collagen antibody-induced arthritis (CAIA) mouse with 1 mm tomographic slices shown from the right paw. **(c) **Quantification of tomographic hind paw fluorescence (picomoles) divided by ankle, midfoot, and toes of arthritic and control mice (n = 12 per group). Study is representative of three separate experiments. **P < 0.001.

To assess the relative benefits of optical imaging of CAIA, we imaged untreated and control mice at the peak of inflammatory disease (day 8) using a three-dimensional FMT imaging approach. In this study, intravenous injection of a cathepsin-activatable agent, ProSense750, allowed the detection of activated inflammatory cells (for example, monocytes, lymphocytes) within the joints and paws of arthritic mice, confirming the findings of other researchers using only semiquantitative two-dimensional surface FRI imaging [33,36,39]. We built upon these earlier observations by assessing three-dimensional FMT imaging and quantification of arthritic mouse paws (in units of picomoles rather than relative fluorescence units), revealing quantitative 30-fold to 40-fold increases in the level of fluorescent signal as compared with control mice (Figure 5a, b).

FMT imaging offered clear advantages in the depth of detection throughout the paw and ankle, as shown by tomographic slices and the ability to clearly establish a pattern of disease with a significantly larger region of inflammation in the ankles than in the rest of the arthritic paw (Figure 5b). Importantly, non-invasive tomographic fluorescence imaging not only detected the inflammation based on increased protease activity, generating three-dimensional tissue images and tomographic slices, but also provided accurate quantification of this fluorescence (Figure 5c). FMT measured a >40-fold increase in ProSense750 fluorescence in the ankles and midfoot of arthritic mice (~100 pmol/ankle) as compared with those of control mice (<5 pmol/ankle) (Figure 5c). In the toe regions, disease was localized to a smaller anatomical area, showing less overall signal and only ~20-fold increased over controls in the toe regions of the hindpaws.

### Optical tomographic imaging of CAIA mice treated with DMARD and non-DMARD therapeutics

As clinical scoring, paw swelling, and plasma biomarkers were not very effective at detecting the differences between DMARD and non-DMARD therapeutics when compared with the histological assessment of inflammation, we used our NIR imaging agents to detect, image, and quantify the protease activity associated with the inflammatory cells in the affected tissue. Such an approach, by virtue of direct labeling of the inflammatory cells, should provide a superior means of assessing therapeutic efficacy comparable with that obtained by histopathology scoring. To achieve this, the study subjects described in Figure 1 were injected intravenously with ProSense750 and MMPSense680 on day 7 for imaging on day 8.

**Figure 6:**
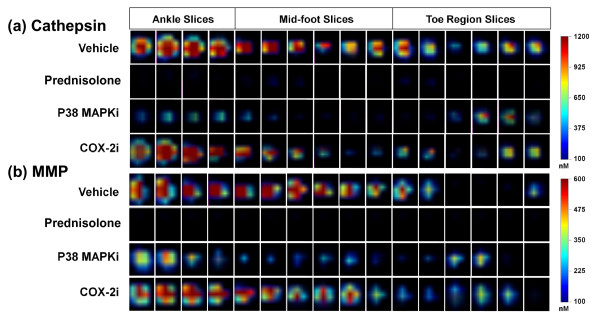
**Near-infrared tomographic slices showing protease activity in paws of arthritic and treated mice**. Representative paws of mice from each of the groups of the study represented in Figure 1 (that is, selected paws at or near the group mean) were analyzed at the level of individual tomographic slices to determine the pattern of fluorescence signal in the ankle, mid-foot, and toe region for each mouse. Color scale represents local regions of fluorescence intensity (nanomolar) concentration. **(a) **Axial tomographic slices of fluorescence resulting from local cathepsin activity. **(b) **Axial tomographic slices of fluorescence resulting from local matrix metalloprotease (MMP) activity. COX-2i, cyclooxygenase-2 inhibitor; MAPKi, mitogen-activated protein kinase inhibitor.

Tomographic slices of paw imaging acquired by FMT showed a high fluorescence signal in vehicle-treated mice as compared with those of controls (Figure 6a, b), and, in support of our contention, the different classes of therapeutics showed differential effects on overall paw fluorescent signal. Prednisolone treatment, as a positive control, showed a clear ablation of both cathepsin and MMP signals across all tomographic slices through the ankle, midfoot, and toe regions, suggesting the complete absence of disease. Similarly, the p38 MAPK inhibitor dramatically decreased the signal throughout most of the paw, with higher apparent effects on the ankle and midfoot than in the toes. In contrast to the effects of the p38 MAPK inhibitor, celecoxib treatment had no obvious effect on the cathepsin and MMP signals in any paw region. Tomographic imaging - in contrast to clinical scoring, paw swelling and plasma biomarker measures - thus discriminated effectively between p38 MAPK inhibitor and celecoxib treatments, and revealed the paw subregions showing predominant therapeutic impact.

### Correlation of quantitative tomography, clinical scores and paw swelling with histopathology

**Figure 7:**
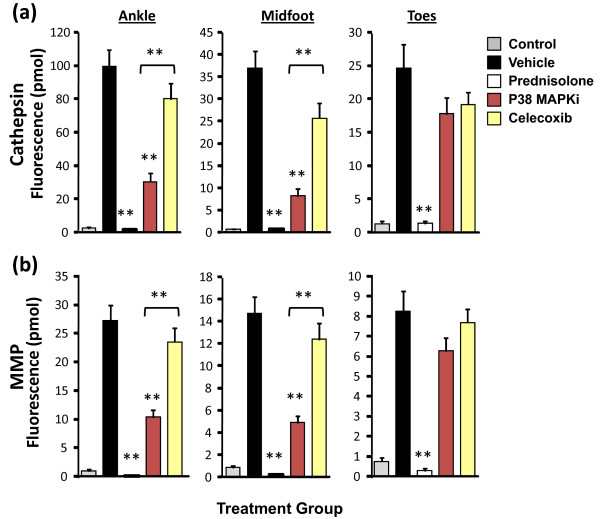
**Near-infrared tomographic quantification of paw subregions of arthritic and treated mice**. Each individual paw (n = 24 per group) from the study represented in Figure 1 was analyzed to quantify the fluorescence signal in the ankle, mid-foot, and toe region for each mouse. **(a) **Tomographic quantification of regional fluorescence resulting from cathepsin activity. **(b) **Tomographic quantification of regional fluorescence resulting from local matrix metalloprotease (MMP) activity. Analysis representative of six studies using a variety of imaging agents. **P < 0.001. MAPKi, mitogen-activated protein kinase inhibitor.

Tomographic quantification of ankle, midfoot, and toe fluorescence in treated and untreated animals showed clear and statistically significant differences in the ProSense750 signal (Figure 7a) and the MMPSense680 signal (Figure 7b) in the ankle and midfoot regions upon p38 MAPK inhibitor treatment, confirming the visual differences in tomographic slices (Figure 6). Only prednisolone showed efficacy in the mild inflammation of the toe region of the arthritic mice. Quantification of imaging datasets revealed an excellent ability to distinguish between a p38 MAPK inhibitor and celecoxib treatments when compared with histopathology (Figure 3a).

**Figure 8:**
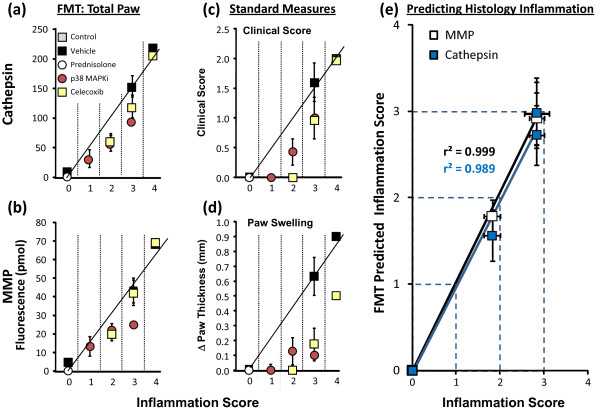
**Near-infrared tomography correlation with histology inflammation scoring**. The study results from individual paws from each group in the study (n = 12 per group, one paw per animal) represented in Figure 7 (using the total of the paw fluorescence) were sorted according to histology inflammation score (0 to 4), and means of the sorted groups were calculated for clinical score, paw swelling, and fluorescence molecular tomography (FMT) imaging readouts. Correlation with histologic inflammation: **(a) **total paw cathepsin, **(b) **total paw matrix metalloprotease (MMP) activity, **(c) **clinical score, and **(d) **paw swelling. **(e) **Non-invasive FMT picomole values were used to predict the pathologist's histology inflammation scores based on the linear relationships between ProSense750 and MMPSense680 to individual paw histology inflammation scores. Results presented as group means ± standard error of the means. MAPKi, mitogen-activated protein kinase inhibitor.

To better understand which CAIA readouts align best with the underlying inflammation, the readouts for individual paws from animals in each treatment were clustered according to their histopathology inflammation scores (from 0 to 4). For each cluster, the average values from optical tomography (cathepsin, MMP), clinical scores, and paw swelling were determined and graphed in comparison with inflammation scores (Figure 8a to 8d). Excellent linear relationships with inflammation scoring were seen with total paw cathepsin (Figure 8a) and MMP (Figure 8b) activity quantification, suggesting that tomographic imaging truly detected and quantified underlying inflammation. Indeed, the non-invasive FMT results (with either ProSense750 or MMPSense680) were able to accurately predict the histology scores determined by the pathologist (Figure 8e).

Neither clinical score (Figure 8c) nor paw swelling (Figure 8d) correlations showed adequate alignment with the inflammation scores, with the pattern of deviation from linearity providing clear evidence that these readouts grossly overestimate drug efficacy regardless of therapeutic class. In addition, it is not surprising that plasma biomarkers showed very poor, nonlinear relationships to inflammation scoring, underestimating or overestimating drug efficacy depending on the biomarker assessed (data not shown).

### Fluorescence molecular tomography quantification of bone changes in CAIA

**Figure 9:**
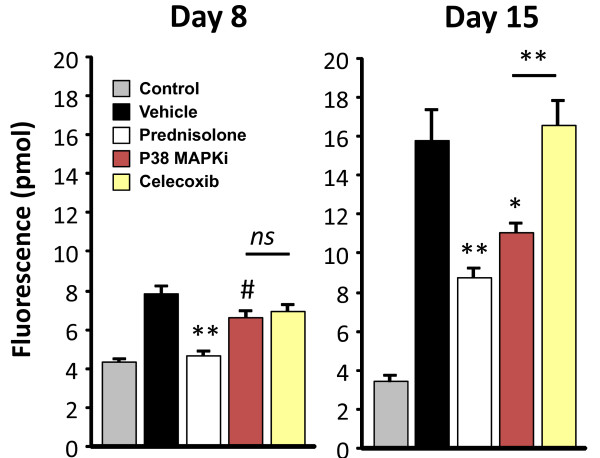
**Tomographic imaging of bone changes in collagen antibody-induced arthritis mice**. Vehicle, control, and treated mice were injected intravenously with OsteoSense680 on day 7 or day 14 and were imaged by FMT2500™. Quantitation of tomographic hind paw fluorescence (picomoles) of arthritic and control mice (n = 6 per group) is represented for day 8 and day 15 imaging. Study is representative of two separate experiments. ^#^*P *< 0.05, *P < 0.01, **P < 0.001; *ns*, not significant. MAPKi, mitogen-activated protein kinase inhibitor.

To assess whether quantitative FMT imaging can detect subtle bone changes in this acute model of arthritis, we imaged mice using OsteoSense680, a NIR-labeled bisphosphonate agent that localizes *in vivo *to hydroxyapatite that is exposed during bone turnover processes. This agent has been used to detect and quantify bone morphogenic protein-2-induced bone growth and fracture repair [40], as well as bone loss in ovariectomized mice [41]. Vehicle-treated animals showed approximately twofold higher OsteoSense680 incorporation into bone as compared with nondiseased controls on day 8 (Figure 9). By day 15, OsteoSense680 incorporation further increased to achieve a ratio of fourfold over normal control animals, suggesting that this imaging agent can detect and quantify arthritis progression. Prednisolone treatment decreased OsteoSense680 incorporation into bone relative to vehicle controls on day 8, maintaining this differential profile on day 15 but showing a higher signal. The p38 MAPK inhibitor treatment showed a minimal, statistically significant decrease in OsteoSense680 signal on day 8, with improved efficacy seen by day 15. In contrast, celecoxib showed no signs of activity by this readout at either timepoint. The OsteoSense680 imaging results on day 15 were thus in strong agreement with ProSense750/MMPSense680 imaging on day 7 as regards discriminating between celecoxib and p38 MAPK inhibitor therapeutic efficacy.

## Discussion

Various rodent models of inflammatory arthritis are used in research and drug development, due to their similar pathology and/or pathogenesis to human RA, their ease of use and reproducibility, and their ability to predict drug efficacy in humans. Although rodent models share a number of important morphologic and immunologic features with RA, they progress rapidly and are heavily reliant upon the acute inflammatory response. Despite this, rodent arthritis models have contributed greatly to the overall knowledge of RA and have led to important advances in therapeutic intervention. Yet disease assessment in rodent models relies heavily upon subjective endpoints (clinical scoring and paw swelling) that emphasize the edema component of disease and capture little or none of the molecular processes that drive differential cellular infiltration and/or bone resorption. Given the emergence of new targeted molecular therapeutic agents (reviewed in [42,43]), improved methods for reliably and objectively detecting and quantifying disease and therapeutic responses without sacrificing the animal (real time) are warranted.

Proteases play a central role in the human RA disease process. We therefore reasoned that protease-activatable NIR imaging agents [44,45] could serve as a sensitive means for reporting disease initiation and therapeutic responses. Such agents have been used to detect protease upregulation in a number of disease states, including cancer [46,47], asthma [37,48], atherosclerosis [32,49], and inflammation [33,36,50]. Proof-of-concept studies using a cathepsin-activatable probe for *in vivo *imaging of protease activity associated with RA animal models have also recently been published [36]. A number of issues remain to be clarified, however, including the utility of optical tomography to provide quantitative readouts that discriminate between DMARD and non-DMARD treatments, and a clear and comprehensive comparison of imaging readouts with standard measures of clinical score and paw swelling.

For the first time, we show the benefit of optical tomographic imaging in a mouse model of arthritis and demonstrate that tomography not only can provide a quantitative measurement of disease severity but also can accurately define the scope of disease in the ankle joints versus interphalangeal joints of the hindpaws (Figure 5). The higher signal in the ankle joints probably indicates a greater magnitude of disease rather than a significantly greater severity of disease processes, as more total disease activity would obviously be occurring in the larger ankle joint. The multiplex approach to quantifying two or more biomarkers correlated with the histopathology, and the fluorescence data could be used to accurately predict histology inflammation scores prior to collection of tissue. These results confirmed that imaging of cathepsin activity, MMP activity, and bone turnover is a successful non-invasive diagnostic modality, capable of providing robust measures of disease progression.

Optical tomographic imaging of NIR imaging agents in CAIA has the potential to be used in drug discovery research by virtue of deep tissue penetration, quantitative readout (picomoles rather than light intensity), and the pairing with NIR imaging agents that detect the cellular participants in the underlying disease pathology. Both p38 MAPK inhibitors and COX-2 inhibitors have previously been shown to effectively reduce clinical signs of disease and paw swelling measurements in rodent models of arthritis [6,7,51,52]. COX-2 inhibitors and other NSAIDs are better at providing symptom relief than at altering disease progression [10,11], however, whereas p38 MAPK inhibitors significantly decrease underlying inflammation and bone destruction [4-8]. We confirmed these observations, showing an overestimation of efficacy of celecoxib and p38 MAPK inhibitors on clinical score and paw swelling as compared with effects by histological assessment (Figures 1 to 3).

A large panel of plasma biomarkers (including IL-6, G-CSF, eotaxin, and MMP-3) also failed to accurately characterize therapeutic efficacy, with both overestimation and underestimation of observed responses (Figure 4) depending on the specific biomarker and therapeutic agent. Although it is likely that the failure of many of the tested biomarkers was due to the very acute nature of this model, it does highlight the disconnection, or perhaps delay, of plasma biomarkers relative to vigorous onset of joint inflammation and destruction. This very failure of plasma biomarkers further suggests that direct imaging of the site of disease should generally be a more accurate means of assessing chronic progression as well as acute flares of disease in human RA. In contrast, imaging with either ProSense750, MMPSense680, or OsteoSense680 provided an excellent discrimination between p38 MAPK inhibitor and celecoxib therapies (Figures 6, 7 and 9), and correlated extremely well with histology inflammation scores (Figure 3a) and human therapeutic observations. In preliminary studies, we found a similar dissociation between standard measures compared with imaging and histology in mouse collagen-induced arthritis when mice were imaged on day 50 post immunization (JD Peterson and TP Misko, unpublished observations).

Imaging CAIA mice using OsteoSense680 appears to be a rapid and sensitive means of detecting changes in bone turnover associated with disease progression. It is important to note that bone resorption was mild in this acute model, as assessed by histopathology (Figures 2 and 3), yet OsteoSense680 showed a twofold increase in signal on day 8 and a fourfold increase by day 15, suggesting significant bone changes were occurring. Furthermore, this readout clearly differentiated between p38 MAPK inhibitor and prednisolone as compared with celecoxib on day 15. It is interesting to note that prednisolone decreased the OsteoSense680 signal on day 8 to the level of the control mice; however, on day 15 the signal increased above the control in the absence of any apparent disease. The longer treatment time with prednisolone probably induced some bone loss, as we have seen previously that this dose of prednisolone will indeed cause some bone loss (and increased OsteoSense680 incorporation) in normal mice (JD Peterson and R Rader, unpublished observations).

## Conclusions

Our studies have built upon the advances in rodent arthritis models as well as in imaging technology to establish non-invasive optical tomographic imaging as a robust and accurate means of assessing inflammation and bone changes in arthritis *in vivo*. For the first time, strong correlations can be shown between quantitative imaging and underlying disease as measured by histologic scoring, such that imaging data can be used to predict histological findings. This technology offers a powerful tool for both basic arthritis research as well as for drug discovery and development by enabling the non-invasive monitoring of cellular processes that also drive human RA pathology. FMT thus should provide a rapid means for selecting DMARD-like drug candidates for clinical evaluation. The future may also bring adaptations of this technology and agents for the monitoring of disease progression and therapeutic intervention in human RA.

## Abbreviations

CAIA: collagen antibody-induced arthritis; COX-2: cyclooxygenase-2; DMARD: disease-modifying anti-rheumatic drug; FMT: fluorescence molecular tomography; G-CSF: granulocyte colony-stimulating factor; H & E: hematoxylin & eosin; IFN: interferon; IL: interleukin; MAPK: mitogen-activated protein kinase; MMP: matrix metalloprotease; NIR: near-infrared; NSAID: nonsteroidal anti-inflammatory drug; RA: rheumatoid arthritis; TNF: tumor necrosis factor.

## Competing interests

KOV, SK, and JDP are employees of VisEn Medical. TPL, MM, RR, JTL, MAA, and TPM are employees of Pfizer Global Research & Development (St Louis, MO, USA). Funding of these studies was shared in a collaboration between Pfizer Global Research & Development and VisEn Medical. The research documents the utility of VisEn imaging agents and imaging technology in addressing specific biological questions in arthritis, but VisEn receives no direct financial gain as a result of publication. Pfizer employees have no financial stake in VisEn Medical.

## Authors' contributions

JDP designed, analyzed, and provided oversight for all *in vivo *imaging studies, KOV performed the *in vivo *studies. SK carried out preliminary *in vivo *validation studies. TPL assessed disease pathology in histologic sections and participated in drafting the manuscript. RR made substantial contributions to study conception. TPM helped in the design and interpretation of the studies and participated in drafting the manuscript. JTL performed the cytokine and chemokine multiplex analysis. MAA performed all MMP-3 assays. MM provided valuable input on arthritis study design and prepared drug formulations.
